# Impact of Attending Physicians' Comments on Residents’ Workloads in the Emergency Department: Results from Two J(^o^)PAN Randomized Controlled Trials

**DOI:** 10.1371/journal.pone.0167480

**Published:** 2016-12-09

**Authors:** Akira Kuriyama, Noriyuki Umakoshi, Jun Fujinaga, Toshie Kaihara, Seigo Urushidani, Naoki Kuninaga, Motohiro Ichikawa, Shinichiro Ienaga, Akira Sasaki, Tetsunori Ikegami

**Affiliations:** 1 Department of General Medicine, Kurashiki Central Hospital, Kurashiki, Okayama, Japan; 2 Department of Emergency Medicine, Kurashiki Central Hospital, Kurashiki, Okayama, Japan; University of South Australia, AUSTRALIA

## Abstract

**Objective:**

To examine whether peppy comments from attending physicians increased the workload of residents working in the emergency department (ED).

**Methods:**

We conducted two parallel-group, assessor-blinded, randomized trials at **t**he ED in a tertiary care hospital in western Japan. Twenty-five residents who examined either ambulatory (J(^o^)PAN-1 Trial) or transferred patients (J(^o^)PAN-2 Trial) in the ED on weekdays. Participants were randomly assigned to groups that either received a peppy message such as “Hope you have a quiet day!” (intervention group) or did not (control group) from the attending physicians. Both trials were conducted from June 2014 through March 2015. For each trial, residents rated the number of patients examined during and the busyness and difficulty of their shifts on a 5-point Likert scale.

**Results:**

A total of 169 randomizations (intervention group, 81; control group, 88) were performed for the J(^o^)PAN-1 Trial, and 178 (intervention group, 85; control group, 93) for the J(^o^)PAN-2 Trial. In the J(^o^)PAN-1 trial, no differences were observed in the number of ambulatory patients examined during their shifts (5.5 and 5.7, respectively, p = 0.48), the busyness of their shifts (2.8 vs 2.8; p = 0.58), or the difficulty of their shifts (3.1 vs 3.1, p = 0.94). However, in the J(^o^)PAN-2 trial, although busyness (2.8 vs 2.7; p = 0.40) and difficulty (3.1 vs 3.2; p = 0.75) were similar between groups, the intervention group examined more transferred patients than the control group (4.4 vs 3.9; p = 0.01).

**Conclusions:**

Peppy comments from attending physicians had a minimal jinxing effect on the workload of residents working in the ED.

**Trial Registration:**

University Hospital Medical Information Network Clinical Trials Registry (UMIN-CTR), UMIN000017193 and UMIN000017194.

## Introduction

Emergency department (ED) personnel tend to be highly superstitious. Famous examples include “Friday the 13th” and a full moon causing an increased number of ED visits. Although several studies have investigated whether such associations exist, no definitive conclusions have been reached [1–5].

One universally prevailing superstition rarely documented in the literature involves the belief that once an upbeat or cheery comment such as "Hope you have a quiet day!" is uttered, the ED suddenly becomes busier with an increased number of visits and admissions[6]. In other words, uttering a cheery or peppy comment brings about the opposite effect. Due to this prevailing superstition, residents often perceive this remark to be a jinx. This is unfortunate because it prevents residents and attending physicians from offering positive comments to their colleagues. To our knowledge, no studies have examined this kind of jinx in the ED setting.

Herein, we conducted two J(^o^)PAN (Jinxes or Peps from Attendings) randomized trials to investigate whether peppy comments from attending physicians increase the workload of residents in the ED. If no evidence can be found for such a jinxing effect, it is hoped that residents and attending physicians can begin to have more genuine conversations.

## Methods

Both J(^o^)PAN trials were single-center, parallel-group, assessor-blinded, randomized trials, and were approved by the institutional review board at Kurashiki Central Hospital. These two trials were registered at University Hospital Medical Information Network Clinical Trials Registry (UMIN-CTR), UMIN000017193 and UMIN000017194. Residents looked through this registry site during the lectures about clinical trials and systematic reviews given to them, and some could know of this study conduct. Thus, we decided to reveal the study protocol after the study ended (S1 File and S2 File). The authors confirm that these trials were to be registered. There was no internal or external funding for this study. We followed the CONsolidated Standards of Reporting Trials (CONSORT) guideline (S3 File) [7].

### Participants

Kurashiki Central Hospital is a 1,131-bed tertiary care hospital in western Japan. A total of 63,054 patients, including 9,125 transfers, were examined at the Kurashiki Central Hospital ED in 2014. All postgraduate year two residents at Kurashiki Central Hospital spent two months at the ED as part of a postgraduate training program. Each resident worked at the ED for a two month-period as part of a five-member shift. In these shifts, two residents examined both ambulatory and transferred patients, one examined only ambulatory patients from 9 am to 5 pm, one examined only transferred patients from 2 pm to 10 pm, and the other worked as the night float physician. The inclusion criteria for residents in the J(^o^)PAN-1 trial were that they saw only ambulatory patients from 9 am to 5 pm, and those for the J(^o^)PAN-2 trial were that they saw only transferred patients from 2 pm to 10 pm. Exclusion criteria included working on weekends, national holidays, and days when board-certified emergency physicians were not available as attending physicians.

The attending physicians distributed questionnaires to the residents at the start of their shifts. The questionnaires were composed of the following items regarding the residents’ workloads: 1) number of patients examined during the shift; 2) busyness of the shift; 3) difficulty of the shift; 4) stress felt during the shift; 5) mealtime duration; and 6) fatigue felt at the end of the shift. Difficulty, busyness, stress and fatigue were each rated on a 5-point Likert scale, with “1” indicating the least and “5” the most. All questionnaires were retrieved at the end of the shifts.

### Interventions

At the start of each shift, the attending physicians received an envelope containing a command for the shift. The command was either to give the peppy message “Hope you have a quiet day” to the residents or to not give the message and behave as usual. The messages were given in the preset randomization sequence described below. Consequently, all eligible residents were randomly assigned to receive a peppy message (intervention group) or no message (control group) from the attending physicians.

### Randomization and blinding

The randomization sequence was created using a computerized random number generator.

The knowledge that this study was being conducted was expected to possibly bias the answers to the questionnaire and, in turn, the study results. Therefore, written consent was waived by the institutional review board and no residents were informed of their participation. To avoid the disclosure, only board-certified emergency physicians (SU, NK, MI, SI, AS, and TI) who were sufficiently “senior” to residents were selected, and the trials were conducted only when these physicians were available as attending physicians. The attending physicians were not blinded for logistic reasons. One ED physician (NU) was involved in the central randomization of the message schedule. A secretary (TK) retrieved the questionnaires and provided data regarding the trends of the ED patients, and another ED physician (JF) made the dataset. The other physician (AK) analyzed the data. The last three individuals were masked to message allocation, and the randomization schedule was only revealed after the analysis was complete.

### Statistical analyses

The primary outcomes for each trial were the number of patients that the residents saw during and the busyness and the difficulty of their shift. The secondary outcomes included the stress felt by residents during their shift, mealtime duration, the fatigue felt by residents at the end of their shift, number of all admissions during their shift, and number of all ambulatory and transferred patients who visited the ED during their shift. The Wilcoxon rank-sum test was used in the analysis of visits to the ED, and the Student’s *t*-test was used to compare the other outcomes between the intervention and control groups. To exclude the potential learning effects of the participants or blunting effect of the message over time, we conducted sensitivity analyses on the primary outcomes with the first 10 shifts of each two months. All statistical tests were two-sided with a significance level of p<0.05. All analyses were performed using Stata v.11.2 (StataCorp, College Station, TX, USA).

## Results

**Fig 1:**
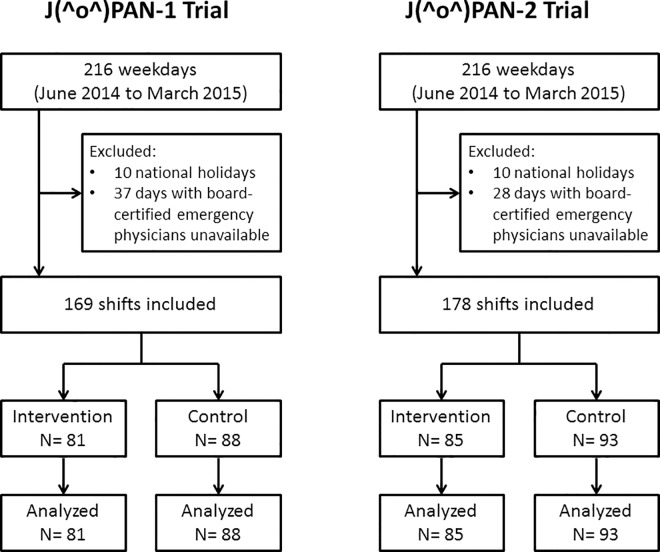
Flowchart of two J(^o^)PAN trials.

Two J(^o^)PAN trials were conducted from June 4, 2014 through March 26, 2015. A total of 25 residents were included, and were followed up only on the relevant day. A total of 169 randomizations were performed for the J(^o^)PAN-1 trial; among the residents, 81 and 88 were assigned to the intervention and control groups, respectively. A total of 178 randomizations were performed for the J(^o^)PAN-2 trial; among the residents, 85 and 93 were assigned to the intervention and control groups, respectively (**Fig 1**). There were 7,517 ED visits (5,495 ambulatory, 2,022 transfers) during the J(^o^)PAN-1 trial session, and 13,276 (10,827 ambulatory, 2,449 transfers) during the J(^o^)PAN-2 trial session. Six board-certified emergency physicians served as attending physicians at similar frequencies between the groups in each trial (**Table 1**). All randomized participants were included in the final analysis, and none were lost to follow-up.

**Table 1:** Characteristics of the Randomizations for J(^o^)PAN-1 and J(^o^)PAN-2 Trial.

	J(^o^)PAN-1 Trial			J(^o^)PAN-2 Trial		
	Intervention group (n = 81)	Control group (n = 88)	P Value	Intervention group (n = 85)	Control group (n = 93)	P Value
**Day of the week**						
Monday	10	17	0.11	17	17	0.88
Tuesday	22	12		19	17	
Wednesday	17	22		15	21	
Thursday	20	17		17	21	
Friday	12	20		17	17	
**Attending physicians**						
SU	7	13	0.20	5	6	0.81
NK	2	5		9	7	
MI	17	14		10	16	
SI	25	22		21	22	
AS	15	25		17	22	
TI	15	9		23	20	

SU, NK, MI, SI, AS, and TI are the initials of the attending physicians.

For the categorical variables, analyses used the Chi-square test.

### Primary outcomes

In the J(^o^)PAN-1 trial, the residents in the intervention and control groups saw an average of 5.5 and 5.7 ambulatory patients, respectively (p = 0.48). The residents in both groups each rated the busyness of their shifts at 2.8 (p = 0.58), and the difficulty of their shifts at 3.1 (p = 0.94) (**Table 2**).

**Table 2:** Summary for Primary and Secondary Outcomes for J(^o^)PAN-1 and J(^o^)PAN-2 Trial.

	J(^o^)PAN-1 Trial			J(^o^)PAN-2 Trial		
	Intervention group (n = 81)	Control group (n = 88)	P Value	Intervention group (n = 85)	Control group (n = 93)	P Value
**Primary Outcomes**						
No. of patients examined (SD)	5.5 (0.24)	5.7 (0.24)	0.48	4.4 (0.16)	3.9 (0.15)	0.01
Busyness (SD)	2.8 (0.11)	2.8 (0.90)	0.59	2.8 (0.09)	2.7 (0.08)	0.40
Difficulty (SD)	3.1 (0.07)	3.1 (0.09)	0.95	3.1 (0.08)	3.2 (0.07)	0.75
**Secondary Outcomes**						
Stress (SD)	2.8 (0.85)	2.9 (0.89)	0.40	3.0 (0.07)	2.9 (0.07)	0.61
Mealtime (SD)	17.2 (1.20)	17.3 (1.10)	0.98	15.6 (1.01)	15.3 (0.98)	0.82
Fatigue (SD)	3.0 (0.10)	3.0 (0.09)	0.85	2.9 (0.74)	2.9 (0.78)	0.96
All Admissions (SD)	10.4 (0.32)	10.6 (0.34)	0.70	14.1 (0.43)	14.0 (0.37)	0.86
All ambulatory patients (IQR)	31 (26 to 36)	32 (24 to 37)	0.98*	53 (46 to 61)	54 (45 to 77)	0.44*
All transferred patients	11.6 (0.38)	12.3 (0.36)	0.19	11 (9 to 15)	12 (9 to 15)	0.68*

Abbreviations; No, number; SD, standard deviation; IQR, interquartile range.

Wilcoxon ranksum test was used for analyses marked with *, and Student *t*-test was otherwise used.

In the J(^o^)PAN-2 trial, the residents in the intervention group saw more transferred patients than those in the control group (4.4 and 3.9 patients, respectively; p = 0.01). The residents from the intervention and control groups rated the busyness of their shifts at 2.8 and 2.7 (p = 0.40), respectively, and the difficulty of their shifts at 3.1 and 3.2 (p = 0.75), respectively (**Table 2**).

### Secondary outcomes

In the J(^o^)PAN-1 trial, no significant differences were found between the intervention and control groups in stress (2.8 vs 2.9, respectively; p = 0.40), mealtime duration (17.2 min vs 17.3 min; p = 0.98), or fatigue (3.0 vs 3.0; p = 0.85) (**Table 2**). In addition, no significant differences were evident in the numbers of admissions (10.4 vs 10.6; p = 0.70) or the number of ambulatory patients that visited the ED (median, 31 vs 32; Wilcoxon p = 0.98).

In the J(^o^)PAN-2 trial, no significant differences were observed between the intervention and control groups in stress (3.0 vs 2.9, respectively; p = 0.61), mealtime duration (15.6 min vs 15.3 min; p = 0.82), or fatigue (2.9 vs 2.9; p = 0.95) (**Table 2**). In addition, no significant differences were apparent in the numbers of admissions (14.1 vs 14.0; p = 0.86) or transferred patients (median, 11 vs 12; Wilcoxon p = 0.68).

No adverse effects were reported.

### Sensitivity analysis

We conducted sensitivity analyses on the primary outcomes for each trial. There was no difference in any outcomes between the groups for each trial, and the point estimates of any outcomes were similar to those in the primary analysis (Table 3).

**Table 3:** Sensitivity Analysis for Primary Outcomes for J(^o^)PAN-1 and J(^o^)PAN-2 Trial.

	J(^o^)PAN-1 Trial			J(^o^)PAN-2 Trial		
Primary Outcomes	Intervention group (n = 23)	Control group (n = 27)	P Value	Intervention group (n = 23)	Control group (n = 27)	P Value
No. of patients examined (SD)	5.2 (2.20)	6.2 (2.22)	0.10	4.4 (1.34)	4.0 (1.60)	0.27
Busyness (SD)	2.9 (1.25)	2.8 (1.08)	0.87	2.9 (0.82)	2.9 (0.73)	0.78
Difficulty (SD)	3.2 (0.13)	3.2 (0.14)	0.95	3.4 (0.58)	3.3 (0.72)	0.62

Abbreviations; No, number; SD, standard deviation; IQR, interquartile range.

Student *t*-test was conducted for all listed outcomes.

## Discussion

The results from our J(^o^)PAN randomized trials suggest that peppy comments from attending physicians have no effect on most resident-oriented outcomes. While the residents in the intervention group examined more transferred patients than those in the control group, the difference (0.5 patients) was clinically small. The total numbers of admissions and ED visits, as well as the shift characteristics, were similar between both groups in either trial. Therefore, peppy comments from attending physicians appear to have little to no clinical impact on the workload of residents and the entire ED. Our findings do not support the existence of such a jinx in the ED setting.

To our knowledge, medical superstitions have only been investigated in two randomized trials [8, 9]. One of these trials included on-call house officers in the United States who were or were not randomly assigned a jinx message stating “You will have a great call day”[8]. Compared with the control group, the “jinxed” group experienced significantly fewer admissions, got more hours of sleep, and reported a lower subjective level of difficulty regarding their work. The other trial, which included on-call medical staffs in Singapore, did not find an association between the consumption of steamed buns (*bao*) by on-call staffs and increased admissions or inpatient mortality[9]. Both trials were conducted in inpatient settings, and their results, similar to those in the present studies, suggested that superstitions had no jinxing effect.

We were concerned that, if the message was repeatedly given to the participants, its novelty and didactic intention might diminish over time, the participants might be suspicious of this study conduct, or a sort of learning effect would occur. We did sensitivity analyses by eliminating the data except for the first 10 shifts, and the analyses produced results similar to the primary analysis (*i*,*e*. the results representing the whole study period). The lack of discrepancy between the primary and sensitivity analyses might suggest that these changes in the participants did not occur during the study.

Murphy's law reads: if something can go wrong, it will, with a probability in direct proportion to the amount of trouble it will cause when it goes awry. There are variants for this law, but all they express perverse outcomes and some superstitions in medicine are derived from this law [10]. If this law did exist and one prayed for the silence in the ED, we had hypothesized that the objective outcomes such as the number of patients visiting the ED and admitted patients would increase. Meanwhile, on the assumption that the residents unconsciously believed in this law, we had presumed that their perception of their workload, such as the busyness and the difficulty of their shift, would be negatively affected by the suggestion of the messages, and be bad ones. Our results suggested that most of the objective and subjective outcomes were similar between the groups. The lack of significant difference in most objective outcomes between the groups suggested that this superstition had no or little, if any, influence in the ED setting.

However, the lack of difference in any subjective outcomes or the residents' perception was surprising. One potential explanation of this finding was the existence of central tendency. The mean values of all residents' perception about the workload were around 3 on a 5-point Likert scale. Central tendency is a famous phenomenon, especially seen in east Asian people, who avoid clear or extreme opinions and prefer to offer ambiguous or "middle" opinions to avoid adverse effects on intra-group harmony, or offence to the questioners or the attending physicians in this case [11–13]. Given that the mean values of all residents' subjective outcomes in either group centrally distributed, it is reasonable that this resulted in the lack of difference in these outcomes.

### Strength and limitations

To the best of our knowledge, our studies were the first randomized trials conducted to examine whether cheery and supportive comments increased the workload of residents in the ED setting. Among trials on medical superstitions, the sample sizes in our studies were the largest to date. The strengths of our studies include the use of central randomization, the assessor-blinded assessment of outcomes, and the fact that no participants were lost to follow-up. All questionnaires were collected at the end of resident shifts, and there was only a small possibility of recall bias for the subjective outcomes.

However, this study did have limitations. First, we did not set the sample size for both trials. Randomized trials of this kind have never been performed in the ED setting, which precluded the sample size calculation. Meanwhile, our trials were considered as pilot studies with small sample sizes, but were conducted for a long period, namely, nearly a year. Thus, we believe that our study provided a pragmatic finding and trend. Second, the association between residents’ baseline level of superstition and their workload was unknown. Some residents perceive themselves as “black clouds,” meaning that they tend to have more severe patients and busier duties than others, who are referred to as “white clouds” [14, 15]. However, questioning the baseline level of superstition would have inevitably biased the answers about residents’ subjective workloads. Furthermore, our trials included a large number of residents, and therefore a small number of randomizations for each resident. These precluded the subgroup analysis regarding whether “black clouds” were destined to face busier duties than “white clouds.” Despite these limitations, our study designs were pragmatic and similar to our usual practice, and therefore, our findings are considered highly generalizable to our practice. Our results could be helpful in promoting a more congenial and thus less stressful working environment for both attending physicians and residents.

## Conclusion

The results from our two J(^o^)PAN trials suggest that peppy comments from attending physicians have minimal to no jinxing effect on the workload of residents working in the ED, or on the entire ED itself. In other words, residents and attending physicians should not be afraid of triggering a jinx when uttering peppy comments such as "Hope you have a quiet day!" Attending physicians should feel free to offer peppy comments to residents if they so choose. Residents should feel confident in accepting such remarks without fear of being jinxed.

## Supporting Information

S1 File Protocol for J(^o^)PAN-1 Trial (PDF)

S2 File Protocol for J(^o^)PAN-2 Trial (PDF)

S3 File CONSORT checklist (DOC)
