# Supplementary material for: Impact of Attending Physicians' Comments on Residents’ Workloads in the Emergency Department: Results from Two J(^o^)PAN Randomized Controlled Trials
Source: PLoS One. 2016 Dec 9;11(12):e0167480. doi: 10.1371/journal.pone.0167480 (PMC5147894; doi:10.1371/journal.pone.0167480)
Supplement: S2 File — (PDF) [file pone.0167480.s002.pdf]

Jinxes or Peps from Attendings Trial 2

J(^o^)PAN Trial-2

Study Protocol

Version 1.1 May 8, 2014
1.2 May 25, 2014

0. Aim

To examine whether peppy comments from attending physicians increased the workload of residents working in the emergency department (ED), literally bringing about the opposite effect.

1. Participants

PGY-2 residents that work at the ED.

2. Rationale

There is a universally prevailing superstition in the emergency department. That is, once a cheery comment such as "Hope you have a quiet day!" is uttered somewhere in the ED, the ED suddenly becomes busier with an increased number of visits and admissions6. Uttering a cheery comment brings about the opposite effect. Due to this prevailing superstition, residents often perceive this remark to be a jinx. We will investigate whether such a jinx exists in the ED; whether peppy comments from attending physicians increase the workload of residents in the ED.

3. Study duration and estimated sample size

Study duration: June 1, 2014 through March 31, 2015

Sample size: at least 150 (A randomized trial of this kind has never been conducted. A sample size calculation is impossible and this trial has a pragmatic nature.)

4. Study Methods

1) Study design

A parallel-group, assessor-blinded, randomized trial

2) Inclusion criteria

PGY-2 resident who will examine only transferred patients from 2 PM to 10 PM

3) Exclusion criteria

PGY-2 residents who will work on weekends or national holidays.

PGY-2 residents who will work on days when board-certified emergency physicians are not available as attending physicians.

4) Methods

The attending physicians will distribute questionnaires to the residents at the start of their shifts. The questionnaires will include the following items regarding the residents' workloads: 1) number of patients examined during the shift; 2) busyness of the shift; 3) difficulty of the shift; 4) stress felt during the shift; 5) mealtime duration; and 6) fatigue felt at the end of the shift. Difficulty, busyness,

stress and fatigue will be each rated on a 5-point Likert scale, with "1" indicating the least and "5" the most. All questionnaires will be retrieved at the end of the shifts.

At the start of the shift, the attending physicians will receive an envelope containing a command for the shift. The command will be either to give the peppy message "Hope you have a quiet day" or "Hope you don't have a busy day" to the residents or to not give the message and behave as usual. The messages will be given in the randomization sequence that is created by Noriyuki Umakoshi with a computerized random number generator (Microsoft Excel). All participants will thus be randomized to receive a peppy message (jinxed group) or no message (non-jinxed group) from the attending physicians.

All residents will be uninformed of this study conduct, because the knowledge might bias the subjective outcomes and therefore the study results. Therefore, the written consent will be waived. Only board-certified emergency physicians (Seigo Urushidani, Naoki Kuninaga, Motohiro Ichikawa, Shinichiro Ienaga, Akira Sasaki, and Tetsunori Ikegami) who are sufficiently older than residents will be selected as the relevant attending physicians. This trial will be conducted only when these physicians are available as attending physicians. Akira Kuriyama is the outcome assessor, who will be completely blinded from the message allocation. The secretary will retrieve the questionnaires and provide data regarding the trends of the ED patients, and Jun Fujinaga will complete the dataset, both of whom will also be blinded from the randomization schedule. This schedule will be revealed after the analysis is completed.

This study protocol will be registered at the UMIN-CTR after all participants complete the study (All participants will participate in the EBM program facilitated by Dr. Akira Kuriyama, and will look through the UMIN.ac.jp and UMIN-CTR for learning about the conduct of clinical researches).

5) Outcomes

Primary Outcomes

① The number of transferred patients that the relevant resident will examine during the shift
② Busyness of the shift
③ Difficulty of the shift

Secondary outcomes

① The number of all transferred and ambulatory patients who visit the ED during the shift (from 2 PM to 10 PM)
② All admissions from the ED during the shift (from 2 PM to 10 PM)
③ The duration taken for supper
④ Pressures/ Stress felt by residents during their shift

⑤ Fatigue/ tiredness felt by residents at the end of their shift

5. Statistical analysis

All outcomes were continuous variables. Student t-test or Wilcoxon runksum test will be used where appropriate to compare outcomes between the jinxed and non-jinxed groups.

6. Adverse effects/ Complication

Attending physicians will cheer up residents, which is common in the daily clinical practice. We don't think this intervention will hurt residents. We will also hear from the residents how they feel during the two-month rotation.

7. Cost related to this study

None.

8. Study conduct

Chief investigator: Akira Kuriyama
Concept of this study: Akira Kuriyama, Noriyuki Umakoshi, Jun Fujinaga
Guarantor: Akira Kuriyama, Tetsunori Ikegami

9. Informed Consent

Written consent will be waived as stated above, because the knowledge might bias the subjective outcomes and therefore the study results. Instead, we will report the study results to all participants. We will also take care of the privacy of the participants: we will separate the identifiable information from the questionnaire sheet as soon as we make the dataset.

10. Presentation of this study results

We will report the study results to all participants. We will also publish this study in a medical journal, with the study results of J(^o^)PAN Trial 1.
